# Letter to the Editor Regarding The Effects of Supraphysiological Estrogen Levels Observed During In Vitro Fertilization Treatment on Cardiac Electrophysiology

**DOI:** 10.1111/anec.70192

**Published:** 2026-04-22

**Authors:** Yongchao Huang

**Affiliations:** ^1^ Graduate School, Guangxi University of Chinese Medicine Nanning Guangxi People's Republic of China

Dear Editor,

We read with interest the recent study by Savaş et al. (2026) which prospectively evaluated electrocardiographic changes during controlled ovarian hyperstimulation in women undergoing in vitro fertilization. The authors should be commended for addressing a clinically relevant and insufficiently studied question. Their paired within‐subject design also provides useful preliminary data on short‐term changes in atrial conduction and ventricular repolarization during the stimulation phase. However, several aspects of the interpretation warrant further consideration.

First, the observed electrocardiographic changes may have been attributed too specifically to estradiol. Although estradiol increased markedly during stimulation, the study design does not isolate estradiol from other concurrent physiological changes related to controlled ovarian hyperstimulation. Several laboratory variables also changed significantly, including luteinizing hormone, sodium, hemoglobin, hematocrit, blood urea nitrogen, creatinine, thyroid‐stimulating hormone, and white blood cell count (Savaş et al. 2026). The authors further note that some of these changes may reflect fluid retention and hemodilution. In addition, the stimulation protocol was not completely uniform, as participants received either recombinant FSH alone or recombinant FSH combined with hMG. Under these conditions, the reported ECG changes may be more appropriately interpreted as reflecting the overall physiological state associated with ovarian stimulation rather than a specific estradiol‐mediated effect.

Second, the mechanistic inference would have been more convincing if the analysis had demonstrated a direct relationship between the magnitude of estradiol change and the degree of ECG change. Although correlation analyses were described in the statistical methods, the manuscript does not report whether changes in estradiol were associated with changes in QTc, Tp‐e, J‐Tp, or PR interval (Savaş et al. 2026). Without such analyses, the argument for estradiol‐specific mediation remains indirect. This point is particularly relevant because the electrophysiological effects of sex hormones are likely to be interactive rather than unidirectional, and may also be influenced by methodological choices such as interval correction (Sedlak et al. 2012; Naderi et al. 2025).

Third, the clinical implications may be stated too strongly. Although QTc and Tp‐e increased significantly, all QTc values remained within the conventional normal range for women, and no overt arrhythmic events were reported (Savaş et al. 2026). Moreover, the study population consisted of otherwise healthy women without obesity, cardiovascular disease, or a family history of sudden cardiac death, whereas the discussion extends the findings to higher‐risk IVF populations. These observations may justify further investigation in such groups, but they do not directly support recommendations for enhanced cardiac monitoring at present.

We commend the authors for drawing attention to the cardiovascular electrophysiology of ovarian stimulation. At this stage, however, the findings may be more appropriately interpreted as evidence of transient ECG interval changes during controlled ovarian hyperstimulation, the clinical significance of which remains to be defined. Further studies incorporating correlation analyses, more homogeneous stimulation protocols, alternative correction methods, and longitudinal arrhythmic outcomes would help clarify whether these changes represent a benign physiological response or a clinically relevant proarrhythmic signal.

Sincerely,

## Author Contributions

The author takes full responsibility for this article.

## Conflicts of Interest

The author declares no conflicts of interest.

## Data Availability

The data that support the findings of this study are available from the corresponding author upon reasonable request.
